# Microplastic pollution on island beaches, Oahu, Hawai`i

**DOI:** 10.1371/journal.pone.0247224

**Published:** 2021-02-18

**Authors:** Savannah Franklin Rey, Janet Franklin, Sergio J. Rey

**Affiliations:** 1 Department of Natural Science, Hawaii Pacific University, Honolulu, HI, United States of America; 2 Department of Botany and Plant Sciences, University of California, Riverside, CA, United States of America; 3 School of Public Policy, University of California, Riverside, CA, United States of America; University of Toronto, CANADA

## Abstract

We report microplastic densities on windward beaches of Oahu, Hawai`i, USA, an island that received about 6 million tourist visits a year. Microplastic densities, surveyed on six Oahu beaches, were highest on the beaches with the coarsest sands, associated with high wave energy. On those beaches, densities were very high (700–1700 particles m^-2^), as high as those recorded on other remote island beaches worldwide. Densities were higher at storm tide lines than high tide lines. Results from our study provide empirical data on the distribution of microplastics on the most populated and visited of the Hawaiian islands.

## Introduction

The pollution of the marine environment by plastic debris is widely recognized as a significant environmental threat globally. Plastics now make up the majority of marine debris [1], and oceans are currently estimated to be filled with trillions of plastic pieces weighing over a quarter of a million tons [2]. Marine plastic debris is harmful to marine organisms and ecosystems; for example, plastic waste is associated with disease in corals [3].

Microplastics are typically defined as plastics 1 μm—5 mm in size (but see [4]). Marine plastic debris can be broken up through oceanic weathering processes into smaller pieces, which is one source of (secondary) microplastics [5, 6]. Other sources of (primary) microplastics are plastic microbeads (in personal care products), as well as plastic resin pellets or nurdles, the raw industrial material for many plastic products, that get into the environment by spillage during handling and transport, and that have been reported on beaches around the world [7]. Microplastics threaten marine [8], terrestrial [9] and aquatic (freshwater) [10] ecosystems worldwide. Microplastics are the predominant form of marine plastic debris [11] and found in marine habitats ranging from the open seas [12], deep oceans [13] and mid-ocean gyres [14], to coastal estuaries [15] and beaches [16].

Microplastics are considered to be particularly harmful to marine ecosystems because they resemble the food of many marine organisms [17], and adsorbed toxic persistent organic pollutants [18, 19]. Small organisms, including those in the beach environment, are key in marine food webs; thus, toxins could be bioamplified. Ingesting microplastics has been shown to affect organisms’ behavior, fitness and abundance [20]. For example, lugworms are ecosystem engineers that oxygenate sediments through bioturbation and are found in intertidal and shallow subtidal muddy and sandy beaches. They have been found to produce fewer casts in sediments with microplastics, indicating less feeding activity [21]. Tosetto et al. [22] found that ingesting microplastics and the associated persistent organic pollutants affected behavior and reduced survival of beachhoppers, a crustacean inhabiting sediments in wave-washed beaches and an important food source for shorebirds.

It has been estimated that between 19 and 23 Mt of “plastic waste generated globally in 2016 entered aquatic ecosystems” [23]. Although mismanaged solid waste in coastal communities is a major source of marine plastic debris [24], ocean current patterns, wind direction, and frequency of beach use are factors that affect distribution of plastic pieces from the oceans back onto shorelines [25, 26]. Microplastics have been found on beaches around the world wherever they have been surveyed, for example on continental beaches in Slovenia [27], Brazil [28], and India [29], and on remote island beaches [30]. In those studies densities typically ranged from tens to hundreds of particles per kilogram or per square meter of sand. Where island beaches have been surveyed their density of microplastics often exceeds continental beaches. In Germany, on nearshore barrier islands in the North Sea [31] an average of 671 particles kg^-1^ was measured, higher than continental shoreline surveyed nearby in Belgium. On remote Easter Island [32] an average of 800 particles m^-2^ was extraordinarily high compared to beaches in mainland Chile.

**Fig 1:**
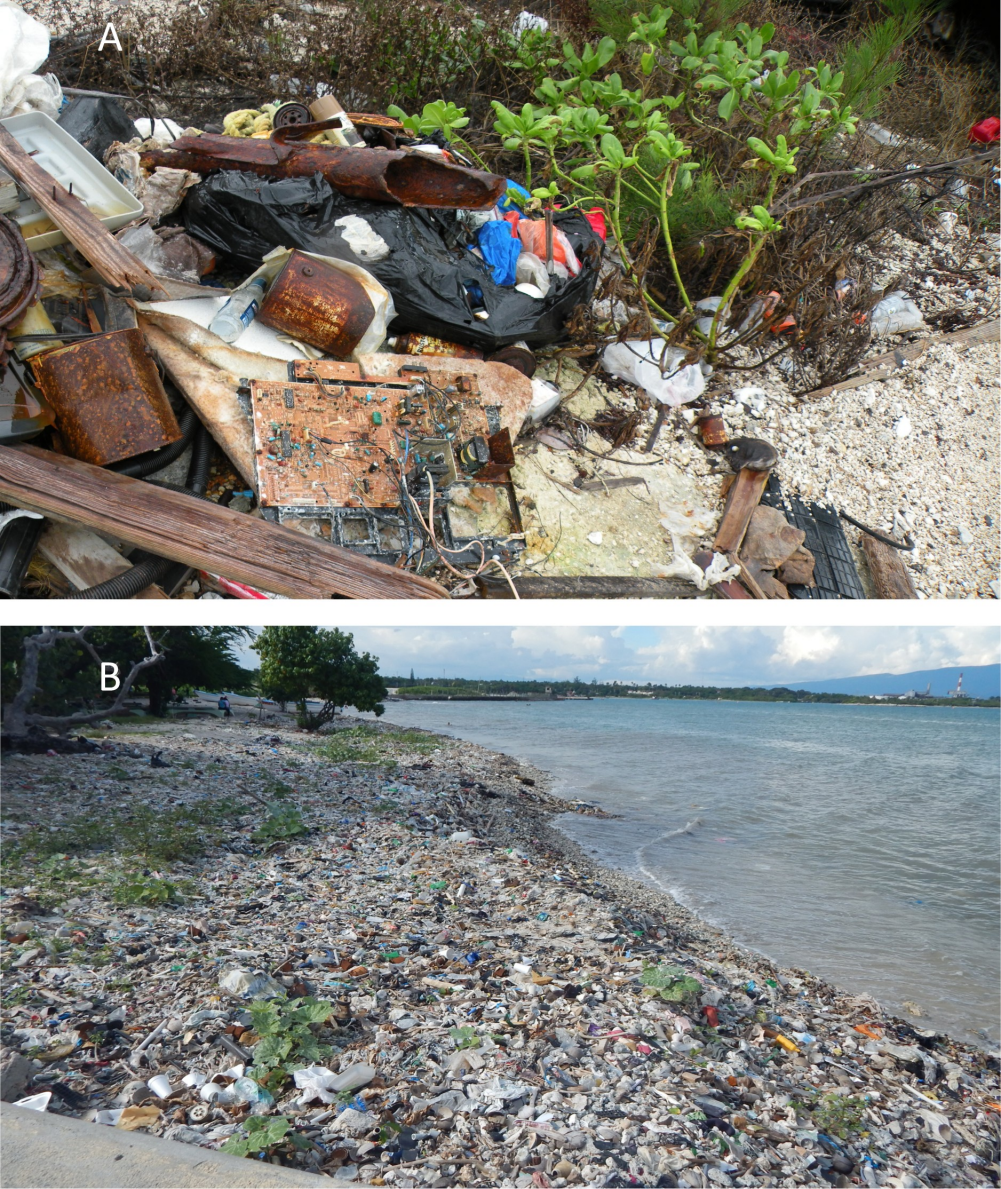
Beach plastic debris on Caribbean islands. Examples of macroplastics and other debris on islands from a) Abaco, The Bahamas (Dec 2017); b) Barahona, Dominican Republic, Hispaniola (Nov 2016) (photos by JF).

Many islands worldwide are the focus of beach tourism, and island beaches often harbor large amounts of debris including plastics (Fig 1). Remote oceanic islands receive plastic debris transported by ocean currents, e.g., in Tonga, the Cook Islands, and Fiji in the southwest Pacific [32], as well as Hawai`i [e.g., 33, 34]. Island beach plastics can also originate from local land-based sources especially where many people do not have waste-removal services or wastewater treatment [35]. A larger proportion of an island, than of a continent, is its coastline (owing to greater perimeter to area ratio for a smaller land mass), and beaches are important locations for recreation and tourism, as well as subsistence and commercial fisheries and other economic activities. Furthermore, beaches–and community beach clean-up events–have become the focus of marine plastics pollution education and outreach in recent decades on continents and islands worldwide [36, 37] (S1 Table).

We present quantitative information on microplastics density and size distribution on six beaches on the island of Oahu, Hawai`i, USA. Oahu is by far the most populated island in the remote Pacific archipelago of oceanic islands, and the focus of a major tourism industry, much of that tourism beach-oriented, drawing visitors from around the world. Oahu is home to more than two thirds of Hawai`i’s 1.42 million people, and hosts more than 60% of Hawai`i’s >9 million visitors per year (https://dbedt.hawaii.gov/visitor/visitor-research/). To our knowledge beach microplastics have not been systematically surveyed in Hawai′i, although important research on beach plastic degradation has been done in Hawai′i [e.g., 25, 33]. We analyzed the density of microplastics on beaches in relation to sand texture (as a proxy for wave energy) and at high tide line versus storm tide line. We expected greater microplastics density on coarse-sand beaches where higher wave energies were expected to deposit more microplastics. We expected to find a greater density of microplastics at storm tide lines where they would accumulate because daily variation in tides would not wash them back into the ocean.

## Methods

### Microplastics survey on Oahu beaches

**Fig 2:**
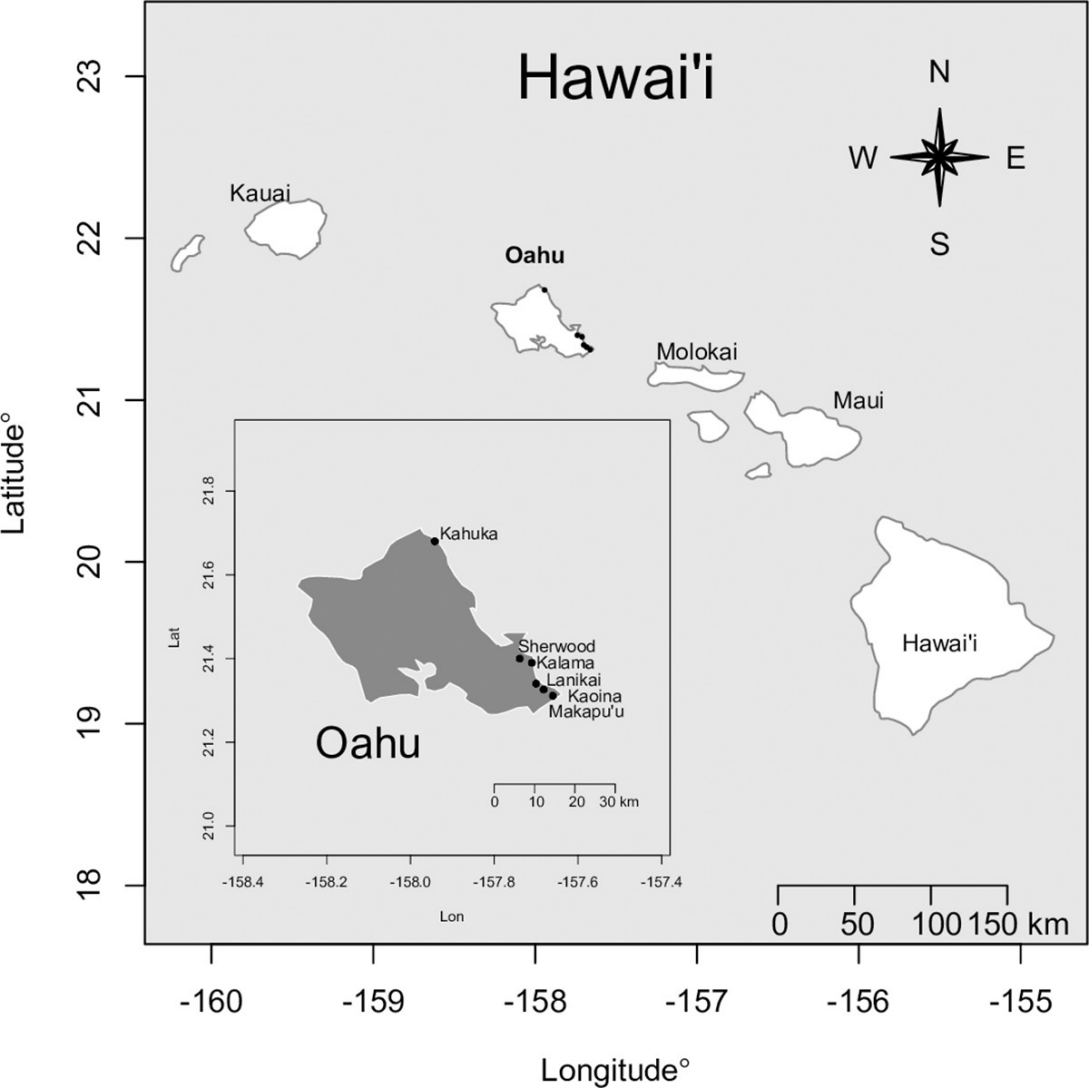
Beaches sampled. Locations of sampled beaches on the island of Oahu, Hawai`i, USA, with inset map showing each beach on the windward side of Oahu: Kahuku, Sherwoods, Kalama, Lanikai, Kaiona, Makapu′u.

We collected microplastics on six beaches on the windward side of Oahu which is subject to constant southeast trade winds and onshore flow (Fig 2). Beaches were initially selected to range from low-wave to high-wave energy using observed beach slope and grain size as proxy. Lanikai and Kaoina beaches have low slopes, fine grained sands and fringing reefs (low-wave energy); Makapu′u and Kahuku are steep beaches with coarse sands (high-wave energy); Kalama and Sherwood are intermediate in their slope and sand texture. We stratified sampling in this way to identify any pattern of plastic density with wave energy. Sand texture was then measured at each beach and microplastic density was analyzed in relation to sand texture (rather than our *a priori* stratification).

**Fig 3:**
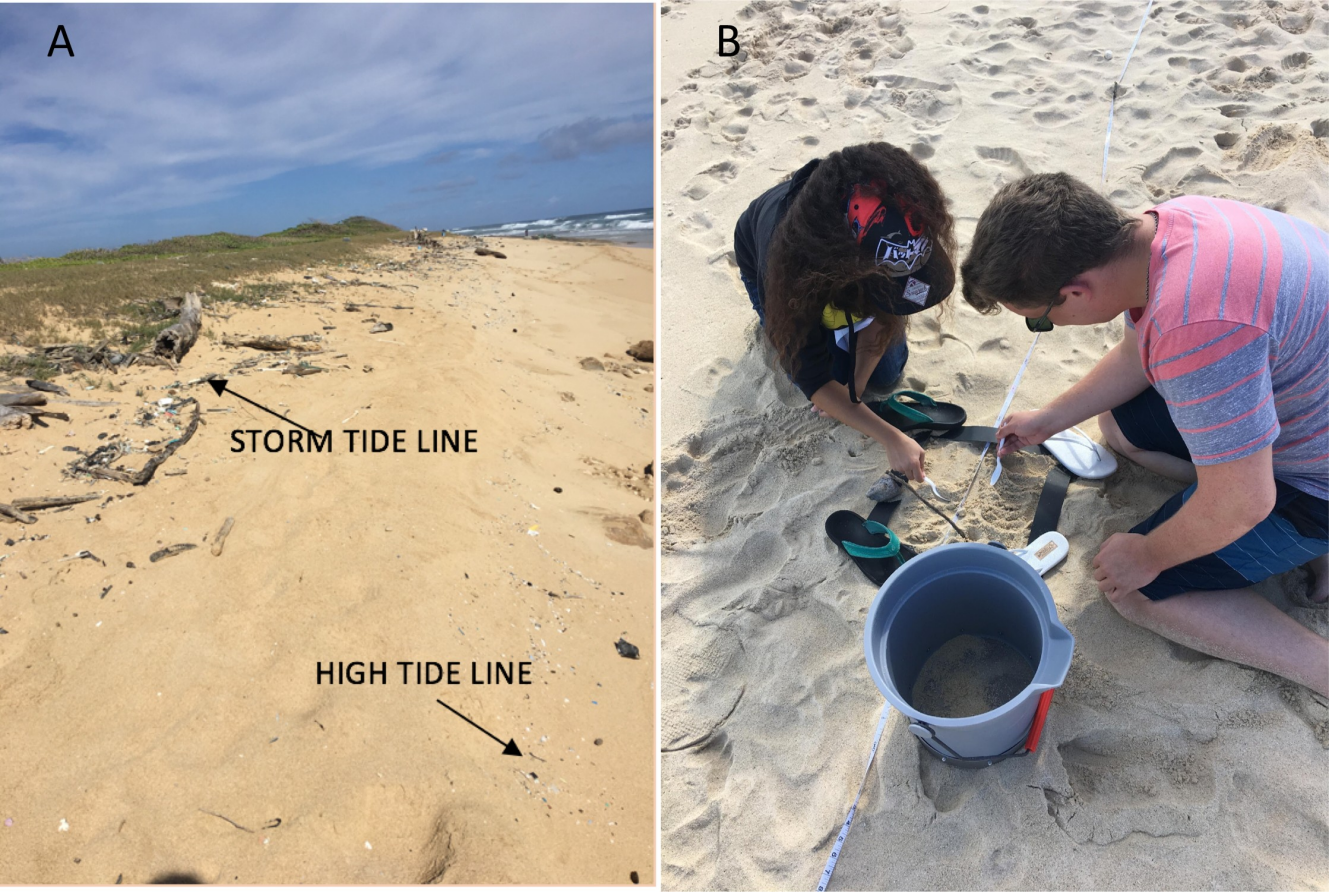
Data collection. Images of A) high and storm tide lines at Kahuku Beach; and B) sampling top 2 cm sand in quadrats at Lanikai Beach.

Sampling was carried out between 1–22 October 2017 (when there were no storm events that would confound microplastics accumulation on different beaches), during low tides. At each beach, the high tide line was identified by a strip of debris or discoloration from the previous high tide, and inland and parallel to the high tide line, the storm tide line was identified by a linear concentration of debris such as branches, twigs or seeds (Fig 3A). A 45-m transect line was haphazardly located [38] in each beach and laid along the high tide line, and storm tide line, in parallel. Random coordinates were generated to locate nine 30 cm x 30 cm quadrats along each transect, and in each quadrat the first two cm of sand [39] was collected (Fig 3B). In this study we quantified microplastics 500 μm—5 mm in size. Larger plastic pieces were separated out. Sand samples were first sieved through a 5 mm then a 500 μm mesh to remove items larger than 5 mm and smaller than 500 μm. In total there were 18 samples from each beach, nine each from high and storm tide lines. Bulk sediment samples were reduced by pouring samples through a 5 mm sieve, discarding larger materials caught in the sieve, and retaining materials that passed through [39].

We minimized self-contamination of microplastic samples during data collection and processing by avoiding fleece and other synthetic clothing, using non-plastic or new (not weathered or worn) equipment, and sampling each beach using same people and equipment. Samples were processed in a laboratory free from sources of plastic particles. Our samples may nonetheless suffer from some level of undetected self-contamination. Self-contamination in microplastics research, however, is usually in the form of very small microplastics, i.e. microfibers [40], from clothing and other sources that may be airborne in the environment, including the laboratory. By restricting our survey to microplastics > 500 μm, we therefore mitigated the effect of contamination on our density estimates and our analysis.

We used the commonly used approach of visually inspecting the reduced sample (in a glass petri dish) with a stereomicroscope (20X/40X) to separate plastics from organic (shells, sand, wood) and inorganic (metal) materials [39]. Criteria used to visually identify microplastics based on their color, shape and texture were: no cellular or organic structures visible; clear and homogenous colors throughout (blue, green, yellow, etc.); particles with uneven, sharp, crooked edges; uniform thickness; and fibers same width throughout their length [39, 41]. All sample processing was done by the same person (SFR) using equal search time per sample. Individual pieces of microplastic collected per sample were counted. Previous studies have shown that other i.e. spectroscopic methods can identify small-sized microplastics overlooked using microscopy [e.g., 42], but by restricting our analysis to particles >500 μm we are unlikely to have significantly undercounted microplastics [differences were only significant in the <50 micron size class in 43]. The number of plastic pieces was then divided by the quadrat size to find the density of plastic pieces per square meter. Size distribution of microplastics was characterized using stacked sieve shakers with mesh sizes of 4 mm, 2 mm and 1 mm. Samples collected from the same tide line at the same site were combined. The stack was shaken for 30 s and then the number of plastics in each size range were counted.

One sample of sand was collected at each beach near the transect lines by collecting the first 2 cm to be analyzed for particle size distribution as a measure of texture. Sand samples from each beach were weighed and distributed through the stacked sieve shaker with mesh sizes 4 mm, 2 mm, 1 mm, 500 μm, 250 μm, 100 μm, 63 μm. The sieve shaker was run for 3 minutes. The portion from each sieve was weighed.

Analysis of variance (ANOVA) was used to identify differences in microplastic particle density among beaches and between tide lines, and a Tukey Honest Significant Differences post-hoc test was used to test for significant pairwise differences in the main effects and interactions. ANOVA was also used to test for differences in microplastics size class distribution with beach characteristics and tide line. We tested for correlation between microplastic density and proportion of coarse sand grains using the Pearson product-moment correlation coefficient. Statistical analysis was done in the R environment [44].

## Results

### Microplastics on Oahu beaches

**Fig 4:**
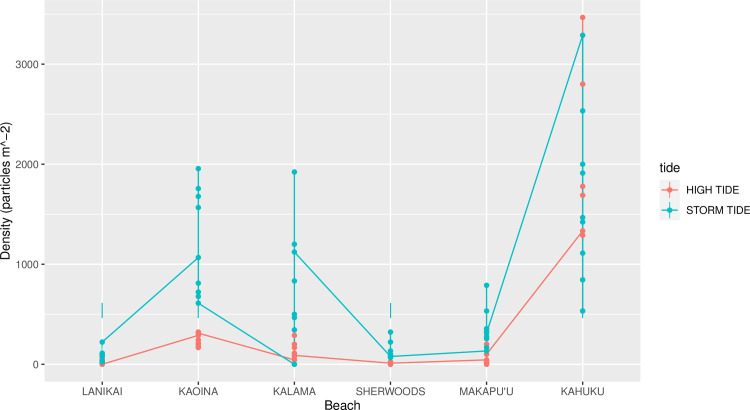
Distribution of microplastic density in transects. Interaction plot showing the distribution of the number of plastic particles (m^-2^) in quadrats for tide line types (N = 54 quadrats per type), and beach (N = 18 quadrats per beach), with the lines connecting the means for each group.

Plastic densities differed significantly among beaches (P<0.001) and between high and low tide lines (P<0.001) according to the ANOVA (and there was a significant interaction, P = 0.005; Table 1). There were higher densities at the storm tide lines than the high tide lines (Fig 4). Plastic densities varied across beaches (Table 1) by an order of magnitude, irrespective of tide line, with the highest average densities at Kaoina and Kahuku (>700 particles m^-2^; Fig 4). The interaction between tide and beach was significant because while there was generally the greater microplastic density at storm tide versus high tide, this difference was mainly driven by higher storm tide densities on Kaoina and Kalama (Fig 4). Storm and high tide densities were similar on the other four beaches, with high densities but also large variances at both high and storm tideline for Kahuku.

**Table 1:** Analysis of variance of plastic particle density* between tidelines and beaches.

**High**	**Storm**				
380.2	693.6				
**LANIKAI**	**KAOINA**	**KALAMA**	**SHERWOODS**	**MAKAPU`U**	**KAHUKU**
52.4	727.2	413.6	70.9	199.3	1757.9
	**Df**	**Sum Sq**	**Mean Sq**	**F value**	**Pr(>F)**
Tide	1	2652367	2652367	12.96	<<0.01
Beach	5	37947650	7589530	37.10	<<0.01
tide:beach	5	3648038	729608	3.57	<0.01
Residuals	96	19641136	204595		

*Plastic particle density (particles m^-2^ in the top 2 cm of sand) among tide lines (high and storm tide line) and beaches.

**Fig 5:**
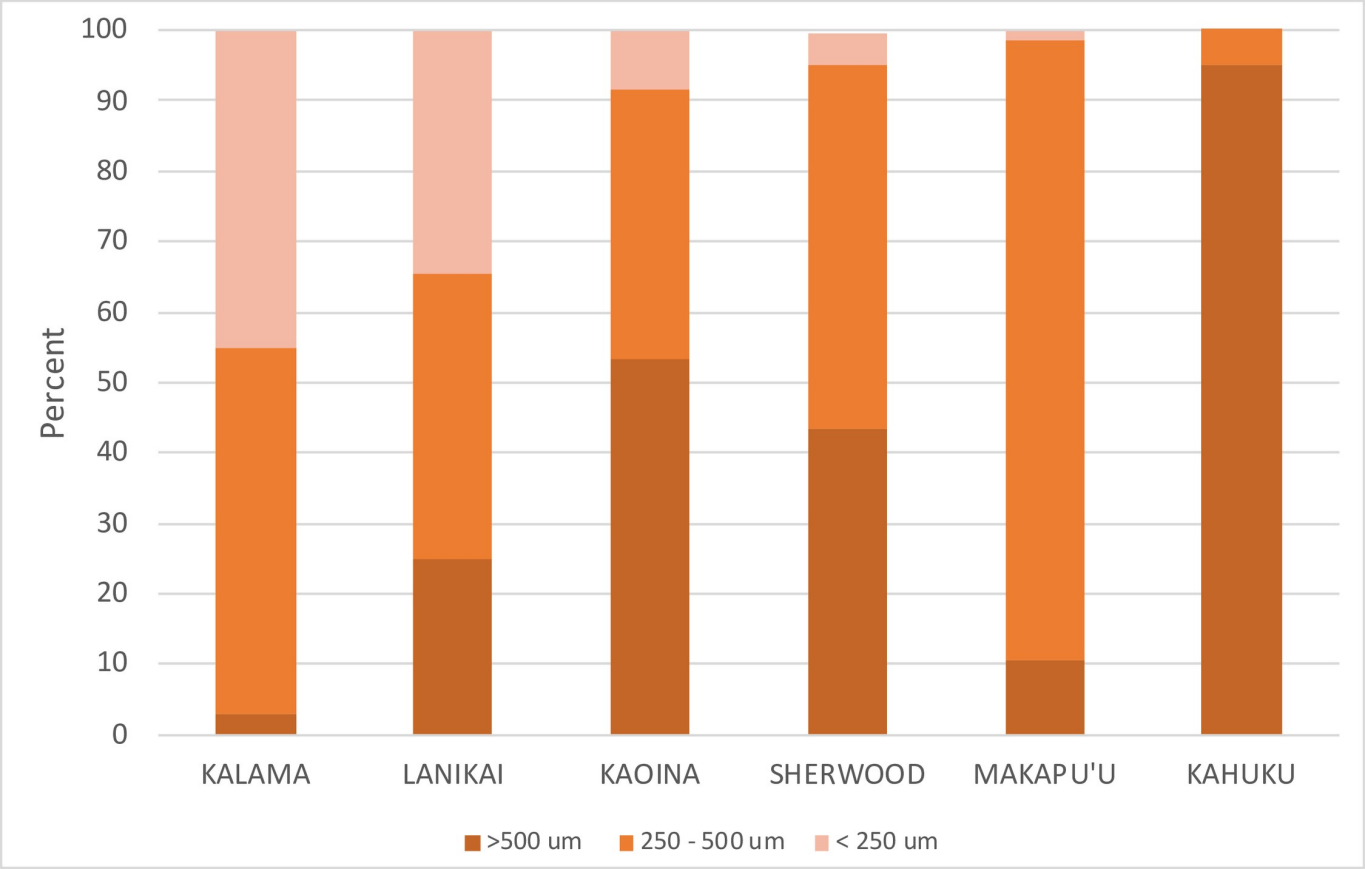
Sand texture on beaches sampled. Percentage composition of sand grain size classes for six beaches arranged from those with more than 30% fine textured sand (Kalama, Lanikai), to those dominated by medium and coarse sands (Kahuku).

Fine textured sands (high proportion of sediment grains <250 μm) were found at Kalama and Lanikai, and coarse (highest proportion >500 μm) at Kahuku. Makapu`u was dominated by the intermediate-sized sediment grains (250–500 μm), while Kaoina and Sherwood were characterized by a mixture of intermediate and coarse with some fine grains (Fig 5). There was a positive correlation between average microplastic density and the coarseness of sand as measured by the proportion of sand in the largest size class for both storm tide line (r = 0.87) and high tide line (r = 0.76).

**Fig 6:**
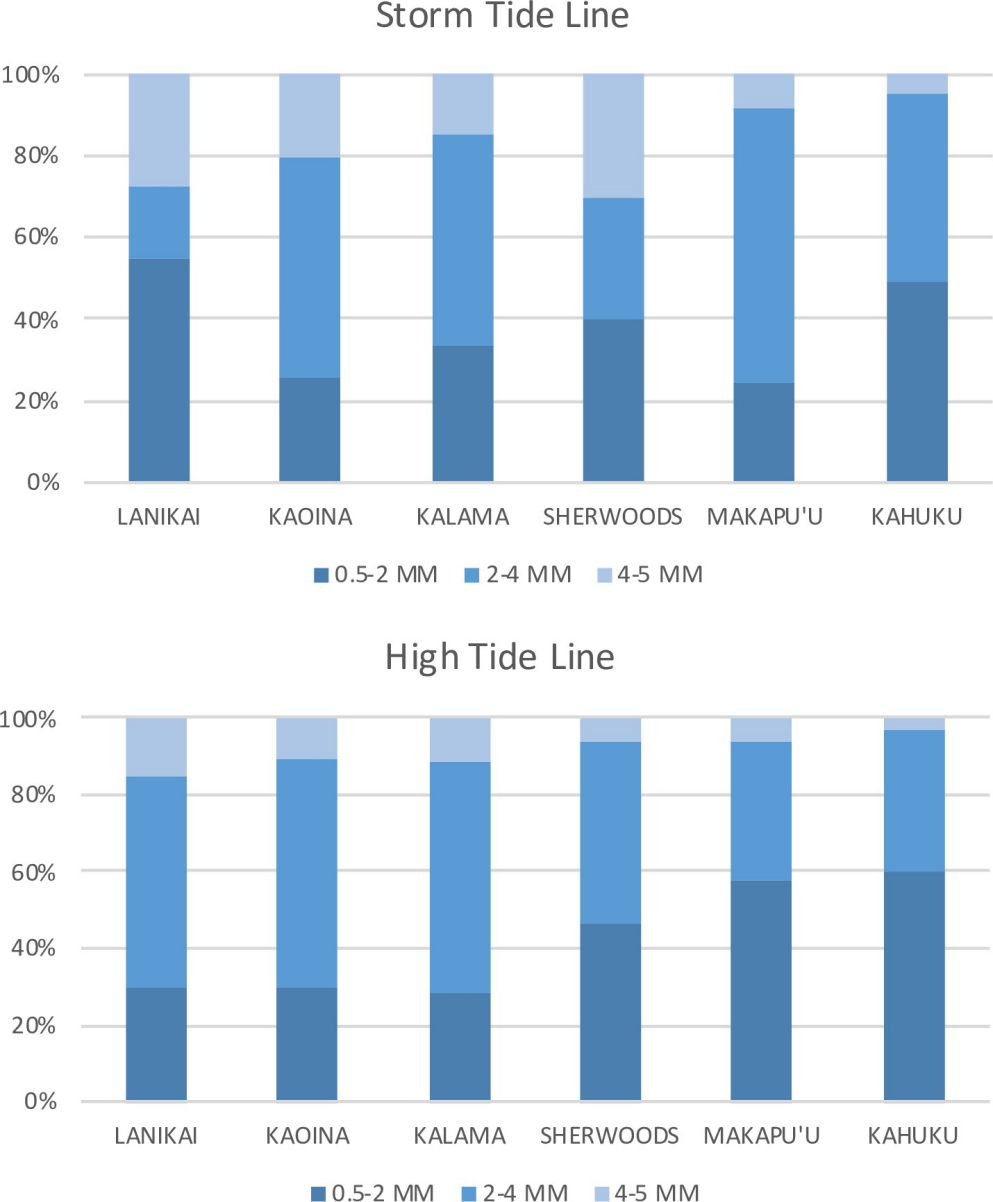
Microplastics size distribution on beaches sampled. Percentage composition of microplastic size classes for six beaches.

The types of microplastics observed [41] were primarily fragments, with some foams, beads and filaments. Most were white, blue, red, yellow, and green. We did not quantity differences in types between beaches or tidelines. The majority (>75%) of microplastics sampled were in the two smaller size classes (<4 mm) that we enumerated (Fig 6). There was no significant difference in size distribution between tide lines, and no relationship between microplastic size distribution and sand texture detected in our study.

## Discussion

Microplastic densities on several Oahu windward beaches were high (>1000 particles m^-2^), comparable to those found on other oceanic islands. Densities were higher at the storm tide than at the high tide, suggesting that wave energy and storm conditions affect density of microplastics found on these beaches. Storm events have been shown to increase microplastic debris both offshore [45] and on beaches, with one study showing an increased number of plastic pellets collected during sampling a few days after a storm [19]. A storm line is formed with the most energetic waves from the previous big wave event. During storm events, especially when they coincide with high tide, energetic waves transport a lot of material from the ocean combined with debris redistributed from the previous high tide line. This could be a contributing factor in the reformation of the high tide line and ultimately the distribution of plastics into the beach environment. Storm events can also cause beach erosion, re-mobilizing buried microplastics that move into the marine compartment and may stay there or return to the beach [46]. While many studies of microplastics on sandy beaches, have focused on high tide line [39], our finding that microplastic densities were higher at storm tide line suggests that sampling storm tide lines in additional to high tide lines may improve estimates of overall microplastics distributions on beaches.

Plastic densities also varied among beaches. Other studies have found great variation in density of microplastics among beaches, as well as seasonal differences [28]. In our study, microplastic density was notably higher on beaches with coarser sand, suggesting that densities are higher on high-wave energy beaches. Factors other than wave energy affect overall density of plastics on a beach. Ocean currents and wind patterns can distribute microplastics to different beaches. The amount of beach use, a factor we could not control in this study, may be a contributing factor to the presence and visibility of a high or storm tide line [19], as well as the amount and sources of microplastics.

Our study only sampled a few locations and provides initial information about microplastics density on Oahu windward beaches. Future research could provide greater detail on the type and source of microplastics, including those in smaller size classes than were considered here, by applying advanced methods such as infrared spectroscopy [47] to more extensive probabilistic sampling, in combination with explicit protocols for separation, contamination control, and identification [39]. Identifying microplastics sources is interesting because on continental beaches or near-shore barrier islands sources are often identified as land-based, coming from wastewater treatment [31] or beach use [29], whereas on smaller, remote, oceanic islands, sources are often exogenous [32, 33]. Future work could also address other factors affecting microplastics distribution on Hawaiian beaches, such as seasonal differences that can result from of rainfall variation or patterns of ocean currents and winds [28]. The effect of land use in Hawaiian beach hinterlands, as well as the amount of beach use, on microplastics amounts and land-based sources would be valuable. This would require more widespread surveys. Nevertheless, our study provides baseline information showing that microplastic densities can be quite high on the popular windward beaches of the most densely-populated and frequently-visited island in the remote Hawaiian archipelago–densities as high as those that have been found on other remote oceanic islands.

## Supporting information

S1 Table Marine microplastics programs. (DOCX)
