# Supplementary material for: Microplastic pollution on island beaches, Oahu, Hawai`i
Source: PLoS One. 2021 Feb 18;16(2):e0247224. doi: 10.1371/journal.pone.0247224 (PMC7891709; doi:10.1371/journal.pone.0247224)
Supplement: S1 Table — (DOCX) [file pone.0247224.s001.docx]

**S1 Table. Marine microplastics programs.** Examples of international, national and regional governmental and non-governmental programs aimed at coastal clean-ups and reducing ocean plastics pollution including (the last three) in islands settings.

| **Scale** | **Organization** | **Type** | **Program** | **Goals; Activities, Initiatives** | **Link** |
| --- | --- | --- | --- | --- | --- |
| International | Ocean Conservancy | Environmental NGO | Fighting for Trash Free Seas | International Coastal Cleanup; Consumer education; Ghost gear; Boating community | [oceanconservancy.org/trash-free-seas/](https://oceanconservancy.org/trash-free-seas/) |
| International | International Union for the Conservation of Nature (IUCN) | Environmental NGO | Close the Plastic Tap Programme | Reduce plastic in oceans by reducing usage and repurposing waste; programs engaging stakeholders in small island developing states, the Mediterranean Sea, Baltic Sea, Thailand, the Azores, and elsewhere with funding from governments and other donors | <https://www.iucn.org/theme/marine-and-polar/our-work/close-plastic-tap-programme> |
| International | Norway | National government sponsoring multilateral (UN, Word Bank), NGO and research organizations | Norwegian Development Program to Combat Marine Litter and Microplastics | Prevent and reduce marine litter and microplastics in developing countries, especially Asian countries with long coast lines, African countries with rapid economic growth, small island developing states; waste management infrastructure; clean-up; prevention | <https://www.regjeringen.no/en/dokumenter/marine_litter/id2642037/> |
| International | 5 Gyres Institute | Environmental NGO | San Francisco Bay Microplastics | Document marine plastics pollution (with a mobile app); education and outreach, influence policy, plastic-free consumer items | <https://www.5gyres.org/> |
| International | Adventure Scientists | Science NGO | Global Microplastics Initiative | Mobilize trained volunteers to monitor microplastics in marine and freshwater systems globally using standard methods (2013-2017) | <https://www.adventurescientists.org/microplastics.html> |
| Multinational | European Environmental Agency | Government | Marine LitterWatch | Data collection by the public using mobile app; partner with community-organized clean-up events at national and subnational scale to collect monitoring data | <https://www.eea.europa.eu/themes/water/europes-seas-and-coasts/assessments/marine-litterwatch#tab-news-and-articles> |
| National (USA) | Surfrider Foundation | Environmental NGO | Rise Above Plastics; Beach Cleanups | Advocate reduction single-use plastics, recycling (Activist Toolkit); Beach cleanups through local chapters emphasizing plastic pollution and data portal for reporting | <https://www.surfrider.org/programs/rise-above-plastics> |
| National | Jamaica Environment Trust | Environmental NGO | International Coastal Cleanup Day - Jamaica | More than 25 years coordinating annual cleanup nationwide, raising awareness about waste management and plastics. (Jamaica has now banned single-use plastics) | https://www.jamentrust.org |
| Subnational (Eleuthera Island, The Bahamas) | Bahamas Plastic Movement | Environmental NGO (local, grassroots) | Plastic Beach Project | Research, education, citizen science, policy change; monitor plastic debris across beaches in South Eleuthera; plastic education camp; upcycle; mentor youth environmental leadership | <http://www.bahamasplasticmovement.org/plastic-beach-project> |
| Subnational (State of Hawai’i, USA) | Sustainable Coastlines Hawaii | Environmental NGO (local, grassroots) | Ocean Plastics Program | Collect beach plastics, recycling, education through marketing, branding | http://sustainablecoastlineshawaii.org |

Examples of programs aimed at monitoring, reducing, and removing marine debris, most with a focus on plastics. NGO = non-governmental non-profit organization; UN = United Nations.
